# Authentication of pork in meat mixtures using PRM mass spectrometry of myosin peptides

**DOI:** 10.1039/c8ra00926k

**Published:** 2018-03-21

**Authors:** Xiao-Dong Pan, Jiang Chen, Qing Chen, Bai-Fen Huang, Jian-Long Han

**Affiliations:** Zhejiang Provincial Center for Disease Control and Prevention Physical-Chemistry Room No. 201 Bin-Sheng Road No. 3399 Binjiang District Hangzhou 310051 China jchen@cdc.zj.cn qingchen@cdc.zj.cn +86-571-87115165 +86-571-87115165

## Abstract

Adulteration of meat products is a major concern not only for economic fraud, but also for ethical reasons. In this study, we presented a parallel reaction monitoring (PRM) mass spectrometry approach for detection of trace pork in meat mixtures (chicken, sheep, and beef). Specific peptides were identified and screened by a shotgun proteomic approach based on tryptic digests of certain protein. Five surrogate peptides from myosin were screened and then used for pork detection by PRM of Orbitrap MS. When the most sensitive peptide was selected, the LOD in mixed meat can be up to 0.5%. The RSD values between detected and designated pork levels (1%, 5% and 50%) were 4–15%. The targeted method developed can be applied to identify and quantify the pork in meat mixture.

## Abstract 2

Adulteration of meat products is a major concern not only for economic fraud, but also for ethical reasons.

## Graphical abstract

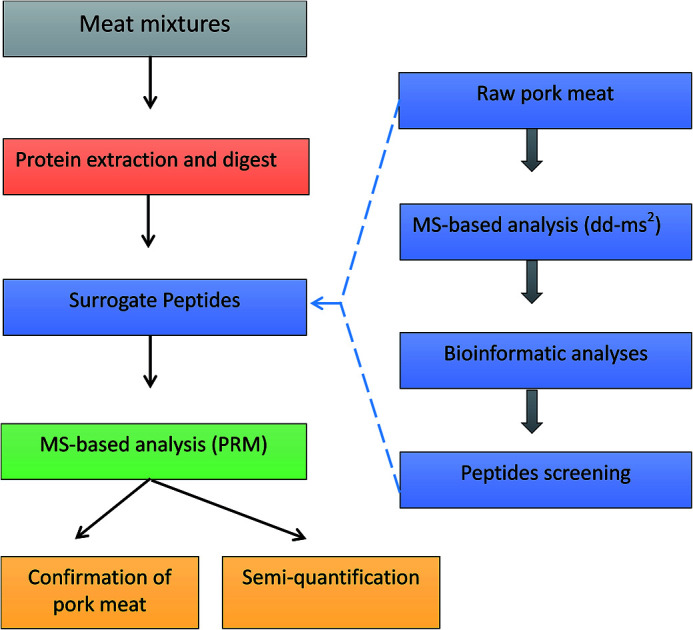

## Introduction

Meat authentication is of great interest for the scientific community, consumers and researchers.^1^ Adulteration of meat products not only misleads consumers but also has ethical and health implications. Consumers have the right to choose the correct meat species on the basis of religious or quality concerns. Although many national and international regulations for labeling food including meat are enforced,^2^ unfortunately, they are not effective towards preventing adulteration. For example, in the European “horse meat scandal” in 2013, at least 50 000 tons of beef meat contained horse meat and 5–7.5% of samples analyzed in the European Union (EU) contained undeclared horse meat.^3^

Reliable analytical strategies for meat authentication are urgently needed to detect trace amounts of meat species. ELISA and PCR are two common methods used for species authentication. However, immunoassays are not exempt from some limitations such as the need for specific antibodies. When antibodies are not highly specific of particular species and/or tissue, it may give rise to false positive cases in terms of cross-reactions.^4^ PCR methods also show some limits, especially in authentication of processed meat. The aggressive conditions in the meat processing such as high temperature and pH change can lead to the disruption of DNA. Another important limitation is that the molecular information obtained is limited and data-mining cannot be performed in post-analysis.^5^

With aims to obtain fast, robust and quantitative methods, various technologies have been considered, such as electrochemical immunosensor, stable isotope ratio (SIR) analysis, NMR spectroscopy, and mass spectrometry based proteomics approaches.^5–7^ Mass spectrometry (MS)-based proteomics methods only quite recently entered the field of food authentication. Specific proteins or peptides can be used as markers for many properties of a food.

Application of mass spectrometry methods to meat authentication has been described recently in the literature.^5^ Our previous study has proved that MS methods (Q-TOF-MS) can authenticate shrimp in fish balls by two specific heat stable peptides from tropomyosin and arginine kinase.^8^ Multiple reaction monitoring (MRM) mass spectrometric method was developed by Watson *et al.* for identification of four kinds of meat (beef, lamb, pork and horse) and detection of one meat added to another at levels of 1% (w/w).^9^ Orduna *et al.* identified proteotypic myoglobin tryptic peptides and characterized meat species by the specific extracted ion chromatograms of Q-Orbitrap MS.^10^ Four marker peptides for processed pork meat were identified by Sarah *et al.* who developed MRM methods for their detection.^11^ Claydon *et al.* constructed a database of heat stable unique tryptic peptides for nine meat species, which could detect down to 0.5% cooked and raw horse in a meat mixture.^12^ Ohana *et al.* used spectral libraries of tryptic peptides to screen 26 different mammalian and bird meats both in raw and processed foods.^13^

However, most of above methods used the MRM or selected reaction monitoring (SRM) for targeted peptides monitoring. Actually, high resolution mass spectrometry, for example Q-Orbitrap MS has the similar analysis mode called parallel reaction monitoring (PRM).^14,15^ It is a targeting MS mode that performs the parallel acquisition of all transitions of the target peptides. This in combination with high selectivity in the quadrupole MS and high-resolution in the Orbitrap MS makes it a good method for targeted proteomics in complicate matrice.^16^ The PRM scanning mode has been applied in several studies, as for the monitoring of species-specific peptide biomarkers to authenticate fish and shellfish species^17,18^ and to quantify the indicated protein in human plasma or serum.^19,20^ However, to our knowledge, the applicability of PRM to the identification and detection of pork in meat mixture still has not been explored.

In current studies, we detected and identified proteotypic peptides of myosin by MS-based proteomic approach. Subsequently, PRM of Orbitrap MS with these peptides was adopted for authentication of pork in meat mixture. The performance of quantification with PRM for the pork proportion was investigated by prepared meat mixture.

## Materials and method

### Chemicals

Ammonium bicarbonate (NH_4_HCO_3_), dithiothreitol (DTT), iodoacetamide (IA) and hydrochloric acid (HCl, 37%) were obtained from Sigma-Aldrich (St. Louis, MO, USA). Acetonitrile (ACN) and formic acid (FA) were purchased from Merck (Darmstadt, Germany). All the reagents used were analytical or HPLC grade. Sequencing grade modified trypsin was from Shanghai Yaxin Biotechnology Co., Ltd (Shanghai, China). All chemical agents were prepared using ultrapure water and without further purification. Ultrapure water was obtained by a Milli-Q Gradient A10 water purification system (Millipore, Bedford, MA, USA) during all the experiments.

### Samples

The commercial fresh meat (pork, chicken, sheep and beef) were purchased from local supermarket (TESCO, Hangzhou, China). Meat mixtures were prepared as follows. Raw pork meat was thoroughly mixed at weight percentage ratios of 1, 5 and 50 with a mixture of equal weight (*i.e.* 1 : 1 : 1) of raw beef, chicken and sheep meat (marked as 1%, 5% and 50% pork meat mixture).

### Bioinformatic analysis

**Fig. 1:**
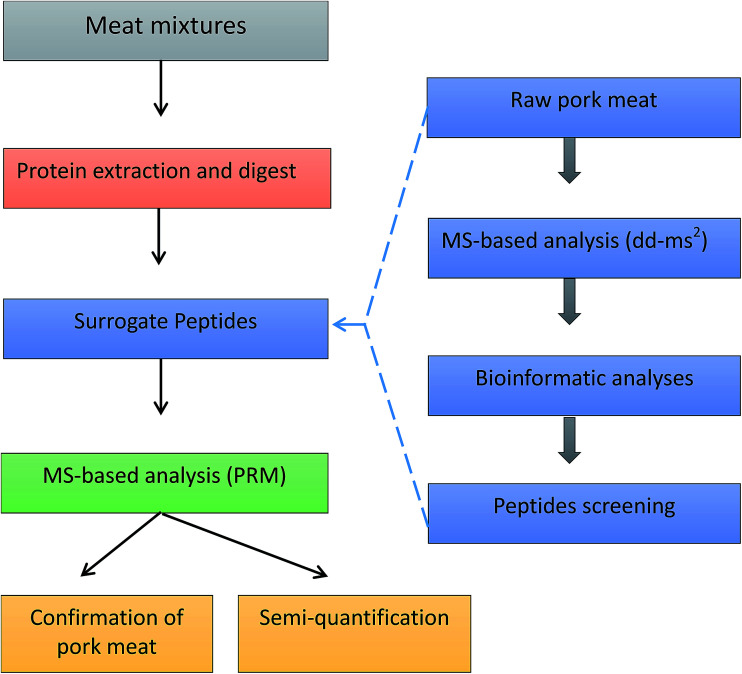
The workflow of pork authentication in meat mixtures.

Protein sequences were obtained from the Universal Protein Resource (UniProt) databases. Protein alignment and sequence comparison were completed by Align tool of Uniprot. Regions of local similarity between sequences were performed by the Basic Local Alignment Search Tool (BLAST) of Uniprot. Protein identification was performed using Thermo Scientific Proteome Discoverer software 2.0 (Thermo Fisher Scientific, San Jose, CA, USA) and MaxQuant software (http://maxquant.org/, version 1.6.0.1). The whole workflow of pork authentication was shown in Fig. 1.

### Sample extraction and digestion

Proteins were extracted from 5 g ground matrix in 15 mL Tris–HCl (200 mM, pH 9.2) by shaking for 30 min. Then, the mixture was sonicated for 20 min at maximum intensity to improve the yield of protein dissolution. The tubs were centrifuged at 5000*g* for 15 min to remove debris. The supernatant was heated at 120 °C for 10 min. Subsequently, the 100 μL supernatant, 100 μL 500 mM NH_4_HCO_3_ and 665 μL deionized water were mixed in an Eppendorf tube. 10 μL 50 mM DTT solution was added to the mixtures and reduced in 40 °C water bath for 30 min at this stage. In the next step an alkylation was performed by adding 10 μL of 150 mM IAA in the dark for 30 min at room temperature. Immediately prior to the incubation, 100 μL of 500 mM NH_4_HCO_3_ and 10 μL of 400 μg mL^−1^ trypsin (freshly prepared) were added and incubated 15 h at 40 °C. The reaction was terminated by addition of 5 μL formic acid. The insoluble substances in tryptic hydrolysates were removed by centrifuging at 13 000*g* for 10 min. Before analysis, the solution was filtered through nylon filters, 0.22 μm × 13 mm (Agela technologies, New York, USA).

### Chromatographic conditions

A Vanquish UHPLC system consisting of a quaternary pump, an autosampler and a column oven was used in this test (all Thermo Scientific, San Jose, CA, USA). Chromatographic separation was carried out on an Acquity UPLC BEH 300 C18 column (1.7 μm, 2.1 mm × 100 mm) maintained at 30 °C. The 0.1% FA aqueous solution (A) and 0.1% FA ACN solution (B) were used for the mobile phases. Gradient elution was: 3% B to 20% B for 2 min; 20% B to 70% B for 14 min; 70% B to 100% B for 1 min; keeping 100% B for 1 min; 100% B to 3% B for 0.5 min; re-equilibration at the initial conditions for 1.5 min for a total run time of 20 min. The flow rate for separations was maintained at 0.3 mL min^−1^ and a 10.0 μL injection volume was used for all standards and samples.

### MS conditions

The UPLC system was coupled to a Q-Orbitrap-MS equipped with a heated electrospray ionization probe (HESI) operating in positive mode (Thermo Fisher Scientific). The following ionization parameters were applied: electrospray voltage 3.5 kV for positive mode, capillary temperature 320 °C, aux gas heater temp 350 °C, sheath gas (N_2_) 40 arbitrary units (arb), auxiliary gas (N_2_) 10 (arb), and S-lens RF level at 50 (arb). The properties of full MS were: full mass resolution, 70 000; dd-MS^2^ resolution, 17 500; stepped NCE, 15, 28, 50; dd settings, charge exclusion, 4–8, >8; peptide match preferred. The properties of PRM were: resolution, 70 000; NCE, 28. The precursor ions and fragment mass used in PRM were obtained by the analysis of full MS/dd-MS^2^ data with Proteome Discoverer software. The instrument was calibrated in positive mode every 7 days using the Pierce LTQ Velos ESI positive-ion calibration solutions from manufacturer (containing caffeine, the tetrapeptide MRFA and a mixture of fluorinated phosphazines ultramark 1621).

## Results and discussion

### Peptide mass fingerprinting

The date dependent acquisition (DDA) of high resolution MS was adopted for collecting peptide mass fingerprinting (PMF) of pork muscle. As described by Kumar *et al.*,^21^ data dependent acquisition is a mode of data collection in tandem mass spectrometry in which a fixed number of peaks selected from a survey scan using predetermined rules, and the corresponding ions are subjected to MS/MS analysis. In Q-Orbitrap, full MS/dd-MS^2^ performs data-dependent scans. Once the targeted compounds are detected, precursor ions that are selected by the quadrupole are sent to the HCD collision cell of the Orbitrap mass spectrometer. In order to get peptide information as much as possible, we set stepped normalized collision energy (NCE) for 15, 28, and 50. The mass spectrometry will perform a stepwise fragmentation on the precursor ion. All fragments created in the steps are collected and sent to the Orbitrap analyzer for one scan detection. In dd-MS^2^ setting, we set minimum AGC target 5.0 × 10^2^ to obtain intensity threshold 1.0 × 10^4^. This field displays the minimum intensity that a mass peak requires to initiate a data dependent scan. The low intensity threshold can record more information of fragments.

### Bioinformatic analysis

Most of previous studies^9,10,22^ explored specific peptides from myoglobin (MB) for muscle authentication. Actually, myosin is also good resource for marker peptide. Myosin comprises a superfamily of ATP-dependent motor protein and is best known for their role in muscle contraction. It is one kind of abundant protein and is plentiful in red or white meat. Furthermore, myosin is relatively stable comparing with other sarcoplasmic proteins.^23^ Accordingly, we selected myosin proteins as the biomarkers for pork muscle. After analysis by Proteome Discoverer and MaxQuant software, main myosin proteins were found (Table 1). Both myosin-4 and myosin-1 owned high scores of Sequest HT (more than 200). Their coverage of peptides in proteins was not less than 30%.

**Table 1:** Myosin from the searching of PMF

Accession	Description	Coverage%	Peptides	PSMs	AAs	MW [kDa]	Score Sequest HT
Q9TV62	Myosin-4	41	70	75	1937	223.1	268.7
Q9TV61	Myosin-1	33	55	57	1939	223.0	205.4
Q29069	Myosin light	49	6	7	150	16.7	26.0
Q19AA7	Myosin light chain	48	7	8	169	18.9	24.6

### Peptide screening

**Fig. 2:**
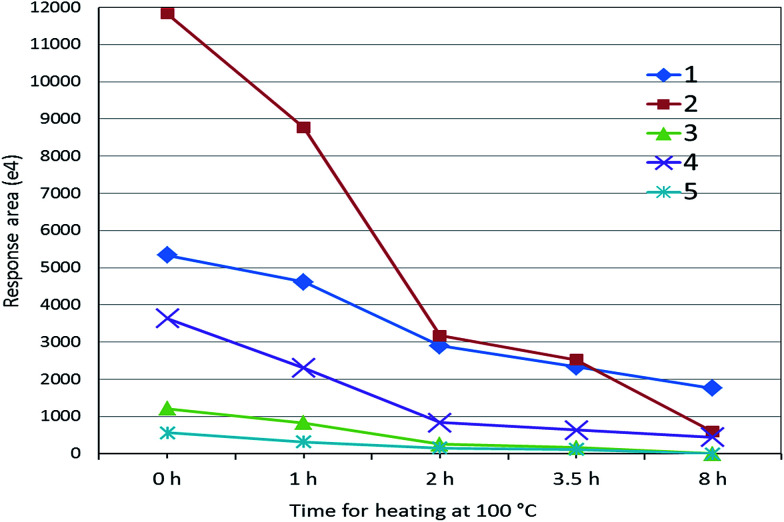
The response of peptides to heating at 100 °C for different times ((1) HKYEETQAELEASQK; (2) KLETDISQIQGEMEDIVQEAR; (3) LETDISQIQGEMEDIVQEAR; (4) KLETDISQIQGEMEDIIQEAR; (5) LETDISQIQGEMEDIIQEAR).

Although 125 peptides for myosin-4 and myosin-1 were identified (Table 1), these peptides cannot be directly used for marker peptides. The screening criteria were: (1) unique or without similarity to related species; (2) easy detected with MS systems; (3) abundant in muscles; (4) reproducible (digestion). After the analysis by extracted ion chromatogram of peptides and BLAST tools, the targeted peptides were founded (Table 2). We selected five peptides (HKYEETQAELEASQK, KLETDISQIQGEMEDIVQEAR, LETDISQIQGEMEDIVQEAR, KLETDISQIQGEMEDIIQEAR and LETDISQIQGEMEDIIQEAR) as the potential surrogate peptides, which are not included in sheep, beef or chicken muscle (Table 2). For evaluating their heat stability, the digested sample of pork muscle was heated at 100 °C for different time. As shown in Fig. 2, mass responses of peptides were all weakened with the increase of heating time. Two peptides of KLETDISQIQGEMEDIVQEAR and HKYEETQAELEASQK can be adopted for authentication on heat-treated meat because of their strong MS response after 2 h heating.

**Table 2:** Myosin-derived marker peptides

Pork	Peptide	Rabbit	Dove	Sheep	Beef	Dog
Myosin-4	HKYEETQAELEASQK	Yes	—			
	NLTEEMAGLDENIAK	Yes		Yes	Yes	
	KLETDISQIQGEMEDIVQEAR	Yes				Yes
	LETDISQIQGEMEDIVQEAR	Yes				Yes
	IAEKDEEIDQMK		Yes			
Myosin-1	KLETDISQIQGEMEDIIQEAR					Yes
	LETDISQIQGEMEDIIQEAR					Yes
Myosin light chain	VLGNPSNEEMNAK	Yes			Yes	Yes

Other peptides of myosin were reported for pork detection by mass spectrometry. Bargen *et al.* used species-specific tryptic marker peptides (TLAFLFAER of myosin-4 and SALAHAVQSSR for myosin-4/-1) for detection of pork by QTrap 5500.^3^ Orduna *et al.* proposed peptide (TLAFLFTGAAGADAEAGGGK of myosin-1) as the specific proteotypic peptide for pork muscle.^10^ But, in present study, these peptides were either not identified or had low MS response, which may be caused by different sample preparation and instrumental conditions.

### Analysis by PRM

**Fig. 3:**
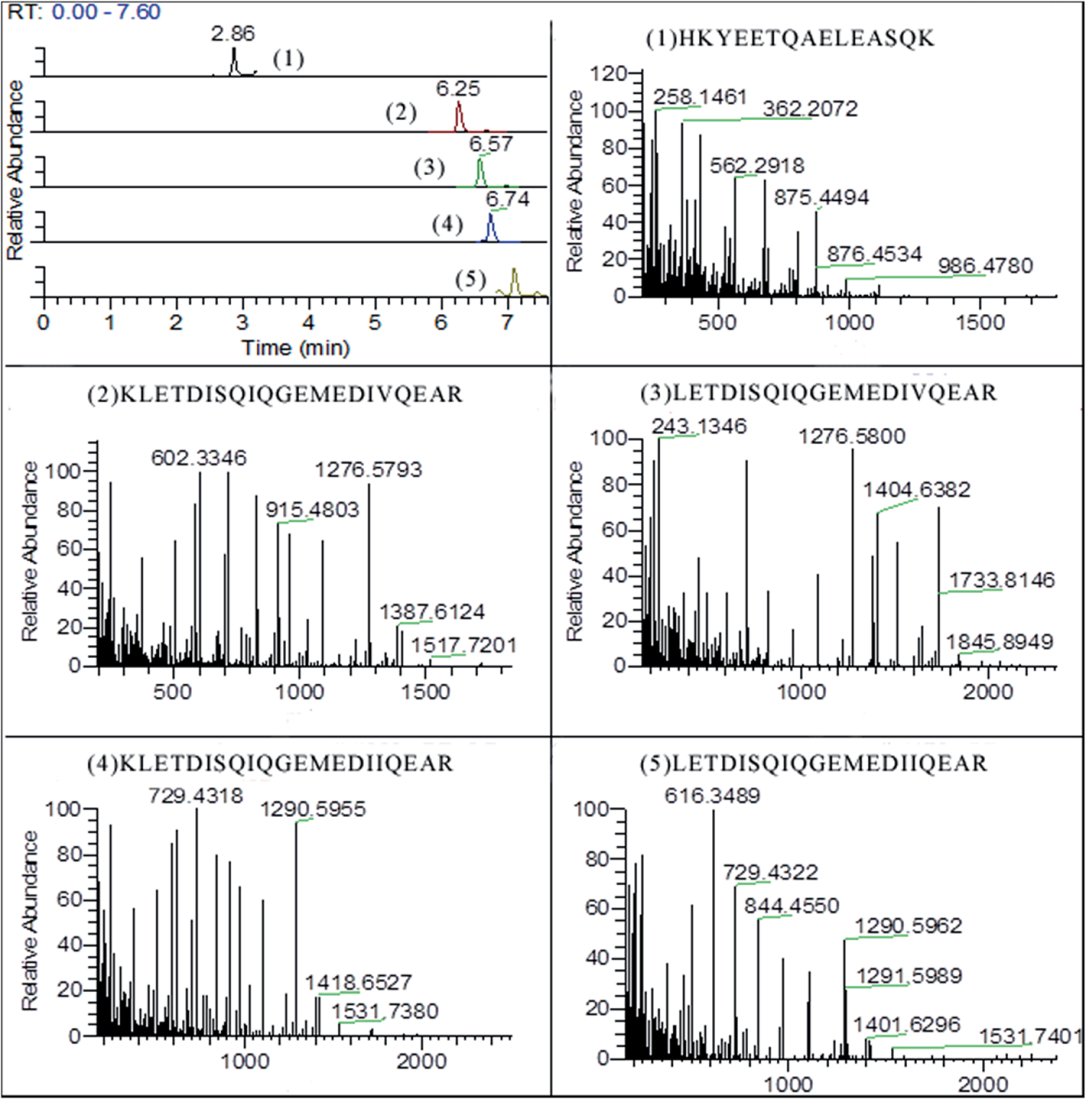
The chromatogram of surrogated peptides and their mass spectra of fragments (5% pork meat mixture).

As shown in Fig. 3, five surrogate peptides were well separated on BEH300 C18 column within 10 min. All fragments of the targeted precursors are measured and the selection of fragments for quantification is done post-acquisition. The identification and quantification were operated with software of Thermo Xcalibur v4.0. The fragmentations of precursor ions were recorded based on b-type and y-type ions which came from cleavage between the nitrogen and carbonyl carbon (Table 3). The y-type and b-type ions can both lose a molecule of water (18 Da) or ammonia (17 Da) in secondary fragmentations. For example, fragments of HKYEETQAELEASQK contained 258.1416 (y2-NH_3_), 986.4780 (y9-NH_3_), 544.2726 (y5-H_2_O), and *etc.* In addition, the b-type ions can lose a molecule of carbon monoxide (28 Da) to give rise to a-type ions.

**Table 3:** Surrogate peptides used for PRM analysis

Peptide	Precursor *m*/*z* (charge)	Main fragments *m*/*z*	RT (min)	Coefficient *R*^2^
HKYEETQAELEASQK	597.6234^(3+)^	362.2072/433.2473/562.2918/875.4494	2.86	0.989
KLETDISQIQGEMEDIVQEAR	811.4039^(3+)^	363.2219/602.3346/715.4158/1276.5798	6.25	0.993
LETDISQIQGEMEDIVQEAR	1152.5548^(2+)^	715.4162/830.4415/1090.5161/1276.5800	6.57	0.905
KLETDISQIQGEMEDIIQEAR	816.0758^(3+)^	503.2656/616.3488/729.4318/1290.5955	6.74	0.967
LETDISQIQGEMEDIIQEAR	773.3775^(3+)^	503.2666/616.3499/729.4322/844.4550	7.09	0.915

In order to explore the linear correlation, pure raw pork muscles (0.5 g, 1 g, 2 g, 3 g, and 5 g) were adopted for obtaining surrogate peptides and analyzed by PRM. A linear regression analysis was calculated by proteotypic peptide peak areas (*y*) and weights of pork muscles (*x*). The correlation coefficient *R*^2^ of five surrogate peptides were all more than 0.9 (Table 3). The limit of detection (LOD) was determined on response at a signal-to-noise ratio (S/N) of 3. When the most sensitive peptide (KLETDISQIQGEMEDIVQEAR) was used, the LOD can be up to 0.005 g pork muscle.

The analyses of the peptides used as surrogates for proteins are commonly performed in mode of SRM or MRM on triple quadrupole mass spectrometers.^24^ In such experiments, predefined series of transitions (precursor/fragment) are sequentially measured for precise quantification. Although it can complete precise quantification over a wide dynamic range, the low resolution of both Q1 and Q3 mass analyzers can result in interfering signals from complicate matrix.^11,25,26^ PRM uses tandem MS to simultaneously monitor fragment ions of the targeted peptide with high resolution and mass accuracy.^27^ The selected precursor ion is isolated by the quadrupole and fragmented in the high-energy collisional dissociation (HCD) cell. The product ions are then monitored by an Orbitrap mass analyzer.^27^ Due to the parallel monitoring, selection of target peptide transitions do not need to be made in advance. Furthermore, PRM offers higher specificity than SRM on QqQ instruments, because it monitors product ions with high resolution, which is less likely to be affected by interfering ions.

### Method optimization and validation

Analytical methods based on peptides strategies rely on the protein extraction and digestion. In extraction step, we adopted sonication to assist the protein dissolution. The extraction solutions were then heated at high temperature of 120 °C in order to inactive protein functions and kill potential microbes. Commonly, targeted proteins for quantification need to be separated by precipitation and SDS-PAGE. This is time consuming and may not be appropriate for rapid analysis of suspecting meat samples. We directly digested the protein in solution without protein purification. A higher temperature (40 °C) was used for protein digestion. All of above procedures were applied in order to improve yield and reproducibility of surrogate peptides.

Peptides-based MS method can perform semi-quantitative and quantitative analysis. For evaluation quantitative results, we prepared meat mixtures of pork meat with beef, chicken and sheep. Meat mixtures (containing pork meat 1%, 5% and 50%) were quantified by the calibration of surrogate peptide peak areas (*y*) and weights of raw pork muscles (*x*). The RSD values (*n* = 5) of 1%, 5% and 50% mixed meat between detected and designated values were 13.8%, 8.5% and 4.6%. The S/N of 1% mixed meat (*n* = 5) was 6–15%, and for 5% mixed meat (*n* = 5) was 30–56%. The relative LOD of this method was 0.5% mixed meat. Actually, studies have indicated that PRM on Q-Exactive instruments and MRM on QqQ MS have comparable analytical sensitivities, dynamic ranges, and precision for protein quantitation.^26,28^ The quantification relies on spiked stable-isotope labeled internal standard (SIS) protein or peptides. However, SIS proteins or peptides were not obtained in present study because of its high cost. We used the raw pork muscles as the external standards for calibrating.

## Conclusion

Following the identification and screening of surrogate peptides, we have developed a sensitive and rapid PRM method based on high resolution Orbitrap MS for pork detection. Using the PRM of targeted precursor for surrogate peptide, the LOD in mixed meat can achieve up to 0.5%. Although the stable-isotope labeled internal standard (SIS) protein or peptides were not obtained, quantitation can be performed using the raw pork muscle as the external standard. The present method can be applied for routine analysis of pork in meat mixtures.

## Author contributions statement

X.-D. P., J. C., Q. C. and B.-F. H. conceived the experiment(s), X.-D. P., and J. C., conducted the experiment(s), X.-D. P., J.-L. H. and B.-F. H. analyzed the results. All authors reviewed the manuscript.

## Conflicts of interest

The authors declare no competing financial interests.

## Supplementary Material
